# Quantification of identifying cognitive impairment using olfactory-stimulated functional near-infrared spectroscopy with machine learning: a post hoc analysis of a diagnostic trial and validation of an external additional trial

**DOI:** 10.1186/s13195-023-01268-9

**Published:** 2023-07-22

**Authors:** Jaewon Kim, Hayeon Lee, Jinseok Lee, Sang Youl Rhee, Jae Il Shin, Seung Won Lee, Wonyoung Cho, Chanyang Min, Rosie Kwon, Jae Gwan Kim, Dong Keon Yon

**Affiliations:** 1 Center for Digital Health, Medical Science Research Institute, Kyung Hee University Medical Center, Kyung Hee University College of Medicine, Seoul, South Korea; 2 Department of Biomedical Engineering, Kyung Hee University College of Electronics and Information, Yongin, South Korea; 3 Department of Pediatrics, Yonsei University College of Medicine, Seoul, South Korea; 4 Department of Precision Medicine, Sungkyunkwan University School of Medicine, Suwon, South Korea; 5 Department of Biomedical Engineering, University of Michigan, Ann Arbor, MI USA; 6 Department of Biomedical Science and Engineering, Gwangju Institute of Science and Technology, Gwangju, South Korea; 7 Department of Pediatrics, Kyung Hee University College of Medicine, Seoul, South Korea

**Keywords:** Cognitive impairment, Alzheimer’s disease, fNIRS, Mild cognitive impairment, Machine learning

## Abstract

**Background:**

We aimed to quantify the identification of mild cognitive impairment and/or Alzheimer’s disease using olfactory-stimulated functional near-infrared spectroscopy using machine learning through a post hoc analysis of a previous diagnostic trial and an external additional trial.

**Methods:**

We conducted two independent, patient-level, single-group, diagnostic interventional trials (original and additional trials) involving elderly volunteers (aged > 60 years) with suspected declining cognitive function. All volunteers were assessed by measuring the oxygenation difference in the orbitofrontal cortex using an open-label olfactory-stimulated functional near-infrared spectroscopy approach, medical interview, amyloid positron emission tomography, brain magnetic resonance imaging, Mini-Mental State Examination, and Seoul Neuropsychological Screening Battery.

**Results:**

In total, 97 (original trial) and 36 (additional trial) elderly volunteers with suspected decline in cognitive function met the eligibility criteria. The statistical model reported classification accuracies of 87.3% in patients with mild cognitive impairment and Alzheimer’s disease in internal validation (original trial) but 63.9% in external validation (additional trial). The machine learning algorithm achieved 92.5% accuracy with the internal validation data and 82.5% accuracy with the external validation data. For the diagnosis of mild cognitive impairment, machine learning performed better than statistical methods with internal (86.0% versus 85.2%) and external validation data (85.4% versus 68.8%).

**Interpretation:**

In two independent trials, machine learning models using olfactory-stimulated oxygenation differences in the orbitofrontal cortex were superior in diagnosing mild cognitive impairment and Alzheimer’s disease compared to classic statistical models. Our results suggest that the machine learning algorithm is stable across different patient groups and increases generalization and reproducibility.

**Trial registration:**

Clinical Research Information Service (CRiS) of Republic of Korea; CRIS numbers, KCT0006197 and KCT0007589.

**Graphical Abstract:**

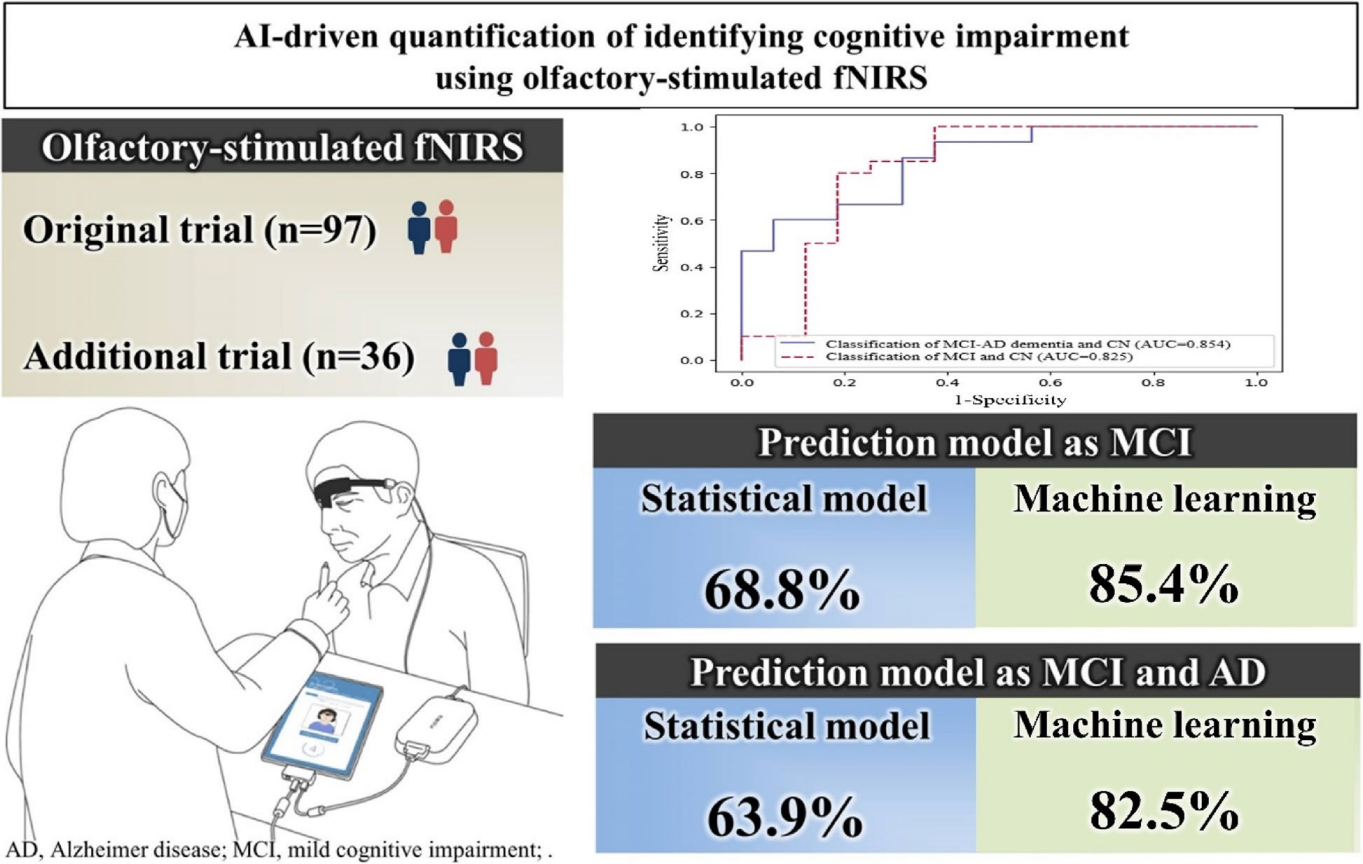

**Supplementary Information:**

The online version contains supplementary material available at 10.1186/s13195-023-01268-9.

## Introduction

Alzheimer’s disease (AD) accounts for 70% of the causes of dementia, and early diagnosis of AD is important to prevent the delay of dementia treatment [1, 2]. Thus, early detection of AD is important for addressing emerging global problems, and previous studies have suggested that olfactory function can be used for the early diagnosis of AD [3–6]. Based on this evidence, we reported a novel approach for the early detection of mild cognitive impairment (MCI) and/or AD dementia using olfactory-stimulated functional near-infrared spectroscopy (fNIRS) diagnostic techniques [7].

Although we suggested a novel approach for the diagnosis of MCI and/or AD Dementia, it is difficult to ensure reproducibility and generalization of this approach in real-world practice. To obtain more reliable AD prediction results, we performed additional trials for independent extra-validation and applied several machine learning algorithms for robust reproducibility and generalization in real-world practice. Through two independent, patient-level, single-group, diagnostic intervention trials, we investigated the potential diagnostic efficacy of olfactory-stimulated fNIRS using machine learning algorithms and quantified this approach through artificial intelligence (AI)-driven fields.

## Methods

### Study design and ethics statements

This study consisted of a post hoc analysis of the diagnostic accuracy trial (total *n* = 97) for which data were published previously [7, 8] and an independent external diagnostic trial (total *n* = 34).

Written informed consent was obtained from each participant and his/her legal guardian at the time of enrollment. The study protocol was approved by the Institutional Review Board of Gwangju Institute of Science and Technology (previous trial, 20210115-HR-58–01-02; additional trial, 20220628-HR-67–02-02). The trial was registered with the Clinical Research Information Service of the Republic of Korea (previous trial, CRIS number: KCT0006197; additional trial, KCT0007589). This study adhered to the tenets of the Declaration of Helsinki.

### Post hoc analysis

The previous study was designed as a prospective, patient-level, single-group, diagnostic accuracy study conducted in 97 elderly volunteers (aged > 60 years) suspected of having declining cognitive function between March 2, 2021, and August 30, 2021. Detailed methods have been described in a previous study [7]. Patients underwent open-label olfactory-stimulated fNIRS to measure oxygenation differences in the orbitofrontal cortex, ^18^F-florbetaben positron emission tomography (PET) amyloid imaging (Discovery STE PET-CT scanner, GE Medical Systems), three-dimensional brain imaging (MAGNETOM Skyra, Siemens Healthineers), apolipoprotein E (*APOE*) genotyping from peripheral blood samples, medical interviews (age, body mass index, sex, education, household income, smoking status, and Charlson comorbidity index [9]), Mini-Mental State Examination (MMSE), Korean Instrumental Activities of Daily Living (K-IADL) [10], and Seoul Neuropsychological Screening Battery (SNSB) [11].

### External additional trial

We additionally included 34 elderly volunteers (aged > 60 years) suspected of having declining cognitive function, with the same inclusion criteria for extra-validation, between July 22, 2022, and August 30, 2022. The trial was conducted by Kolab (Gwangju, South Korea), an International Organization for Standardization-certified International Contract Research Organization. All the same tests (fNIRS, PET, brain MRI, *APOE* genotyping, medical interview, MMSE, K-IADL, and SNSB) as in the original trial were performed.

### Alzheimer classification criteria

The stages of AD were divided into normal, MCI, and AD dementia, and these criteria were divided based on the 2011 National Institute on Aging-Alzheimer’s Association recommendations [12]. Normal cognitive function was defined as patients with normal MMSE or SNSB results, MCI as a *z*-score (normalized for age and education level) <  − 1.0 on at least two cognitive domains of the SNSB tests (memory, attention, visuospatial function, language, and related function, and frontal/executive function) according to the comprehensive criteria of Jak/Bondi [13], and AD as those with MCI and impairments in daily functioning according to the K-IADL.

### Diagnostic procedure

Using the same fNIRS system as in the previous study [7], we measured the activation of the prefrontal cortex during olfactory stimulation (N2; N.CER Co.Ltd, Gwangju, South Korea). It can trace the hemoglobin oxygen concentration in the cerebral cortex over time [14]. In this study, we placed the FP1 and FP2 sides on the upper eyebrow according to the International 10–20 System for electroencephalography (EEG) measurements [7]. One cycle of the olfactory stimulation process was performed before a break of 40 s, followed by stimulation for 20 s (one cycle: 1 min), and then three cycles were conducted (total time: 3 min). Olfactory stimulation was stimulated using a sniffing stick pen (unscented and peppermint scented; Burghart Screening 12 Test) [15].

### Statistical analysis

Baseline data are presented as median and interquartile range or mean and standard deviation. Statistical analysis was performed using R software, version 3.1.1 (R Foundation, Vienna, Austria), and SPSS (version 25.0; IBM Corp., Armonk, NY, USA) [16–18]. Two-tailed *P*-values < 0.05 were considered statistically significant.

We used the following covariates: age, sex, body mass index (< 25 kg/m^2^ [normal] and ≥ 25 kg/m^2^ [overweight or obese]), years of education (continuous variable), household income (low [1–29 percentile], middle [30–69 percentile], and high [70–100 percentile]) [19], smoking status (never or ex-smoker and current smoker), Charlson comorbidity index (0, 1, and ≥ 2) [9, 20], APOE4 carrier, MMSE results, *z*-score (normalized for age and education level) of the SNSB test results (memory, attention, visuospatial function, language and related function, and frontal/executive function), standard uptake value ratio from amyloid PET, and hippocampal volume on brain MRI. We also used C-statistics to express the mean area under the receiver operating characteristic curve (AUC) using 95% confidence intervals as statistics for the predictive model of MCI and/or AD.

### Features of the machine learning models

To validate our proposed machine learning models, we performed fivefold cross-validation for our proposed machine learning models from the previous trial data (*n* = 97). Then, we validated the models using additional external trial data (*n* = 34). In this study, we proposed two machine learning models: (1) classification of MCI and cognitively normal (CN) and (2) discrimination between MCI-AD dementia and CN. The results of our models were compared with those obtained using a previous statistical approach [21]. To ensure a fair comparison between statistical modeling and machine learning, a calibration procedure for the fNIRS values used in the statistical model was also performed in machine learning. To match fNIRS values to the calibration procedure between the two approaches, we extended the following four feature values: fNIRS × years of education, fNIRS × household income, fNIRS × the Charlson comorbidity index, and fNIRS × age. Thus, a total of 11 features were used for the two aforementioned machine learning models.

### Proposed machine learning models

**Fig. 1:**
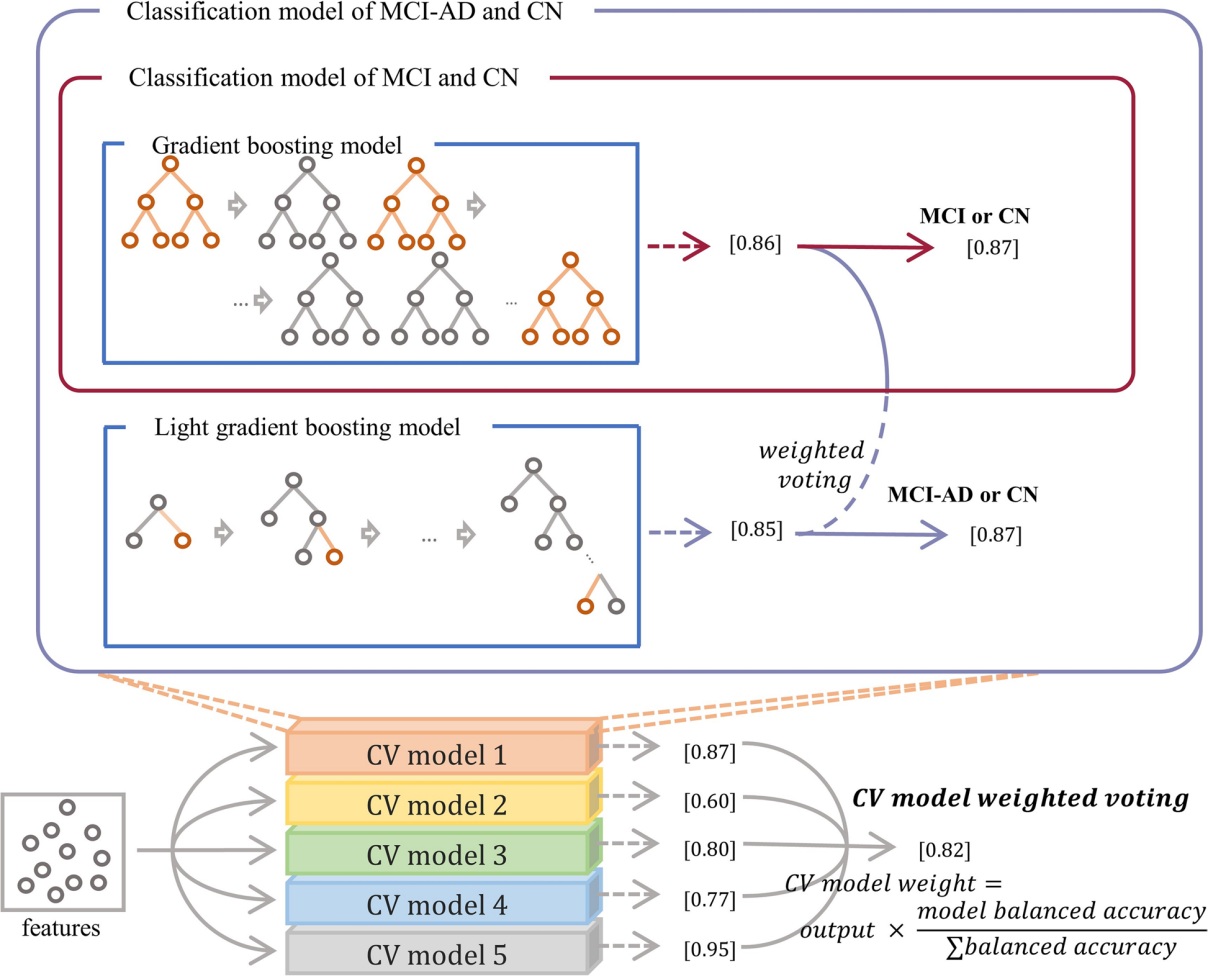
Our proposed overall architecture for the two models: the classification of MCI and CN uses an ensemble approach combining the three models of XGBoost, GB, and LGB, and the classification of MCI-AD and CN uses an ensemble approach combining the four models of XGBoost, GB, LGB, and AdaBoost. The balanced accuracy values from five models via fivefold cross-validation were used for cross-validation model weights to combine the five models. CN, cognitively normal; MCI, mild cognitive impairment; XGBoost, extreme gradient boosting; GB, gradient boosting; LBG, light gradient boosting; AdaBoost, adaptive boosting; AD, Alzheimer’s disease

Figure 1 illustrates the overall architecture of the two machine learning models for classifying MCI, CN, MCI-AD dementia, and CN. For both models, we first computed the values of the mean and standard deviation of each feature from the previous trial data and normalized all feature values from both datasets so that they had zero mean and unity standard deviation.

First, to classify MCI and CN, we used a light gradient boosting (LGB) model, which commonly trains data based on the gradient boosting principle. We applied an exhaustive search (brute-force search) and sequential model-based optimization (SMBO) to determine the optimum hyperparameters of the model. For LGB, we found the following optimum parameters: boosting parameter of gradient-based one-side sampling (GOSS), maximum depth of 4; learning rate, 0.0001; number of tree estimators, 100; fraction of observation, 0.5; fraction of columns, 0.1; and maximum number of leaves, 20. Based on the optimized models, we computed the probabilities of MCI and CN by averaging the outputs from the XGBoost, GB, and LGB models. Subsequently, we computed balanced accuracy values from the five models via fivefold cross-validation and used the accuracy values as cross-validation model weights. By weighting the cross-validation model weights to the probability values derived from the five models via fivefold cross-validation, we obtained the final probabilities for MCI and CN.

Second, for the model to classify MCI-AD dementia and CN, we used an ensemble approach combining GB and LGB models. Here, one additional GB was combined with the model to classify MCI and CN. Similarly, we determined the optimal hyperparameters for each model. For GB, we found the following optimum parameters: maximum depth, 3; learning rate, 0.2; number of tree estimators, 100; and minimum number of observations, 4. For LGB, we found the following optimum parameters: boosting parameter of gradient-boosted decision trees (GBDT) maximum depth, 6; learning rate, 0.25; number of tree estimators, 100; fraction of observation, 0.6; fraction of columns, 0.6; and maximum number of leaves, 33. Based on the optimized models, we computed the probabilities of MCI-AD dementia and CN by averaging the outputs from the GB, and LGB models. Next, we computed balanced accuracy values from the five models via fivefold cross-validation and used the accuracy values as cross-validation model weights. By weighting the cross-validation model weights to the probability values derived from the five models via f-fold cross-validation, we obtained the final probabilities of MCI-AD dementia and CN.

All processing steps were performed on a personal computer equipped with an Intel Core i7-12700F 4.9-GHz CPU, 512 GB of memory, and NVIDIA GEForce RTX 3080 Ti GPU. The models were implemented using Python (version 3.7.13) with TensorFlow-gpu (version 2.6.0), Keras (version 2.9.0), NumPy (version 1.19.5), Pandas (version 1.3.5), Matplotlib (version 3.5.1), and Scikit-learn (version 1.0.2).

## Results

In total, 97 (original trial) and 36 (additional trial) elderly volunteers (aged > 60 years) with a suspected decline in cognitive function met the eligibility criteria. For the overall trial, 133 participants were recruited, of whom 71 (53.4%) were CN (median age 74.0 years; female sex 52.9%), 41 (30.8%) had MCI (median age 74.0 years; female sex 53.7%), and 21 (15.8%) had AD dementia (median age 76.0 years; female sex 47.6%; Tables 1 and S1).

**Table 1:** Baseline characteristics of participants at enrollment (previous trial *n* = 97 and additional trial *n* = 36)

	Total	CN	MCI^a^	AD dementia
Number (%)	133 (100.0)	71 (53.4)	41 (30.8)	21 (15.8)
Age, years, median (IQR)	74.0 (71.0 to 78.0)	74.0 (71.0 to 78.0)	74.0 (68.5 to 77.0)	76.0 (72.5 to 82.0)
Body mass index, kg/m^2^, *n* (%)				
< 25 (normal)	87 (65.4)	46 (64.8)	25 (61.0)	16 (76.2)
≥ 25 (overweight or obese)	46 (34.6)	25 (35.2)	16 (39.0)	5 (23.8)
Sex, female (%)	68 (51.1)	36 (52.9)	22 (53.7)	10 (47.6)
Education, years, median (IQR)	12.0 (6.0 to 14.0)	9.0 (6.0 to 12.5)	12.0 (6.0 to 13.0)	10.0 (6.0 to 16.0)
Household income, n (%)				
Low (1–29 percentile)	30 (22.6)	12 (16.9)	11 (26.8)	7 (33.3)
Middle (30–69 percentile)	58 (43.6)	30 (42.3)	20 (48.8)	8 (38.1)
High (70–100 percentile)	45 (33.8)	29 (40.8)	10 (24.4)	6 (28.6)
Smoking status, *n* (%)				
Never or ex-smoker	127 (95.5)	68 (95.8)	40 (97.6)	19 (90.5)
Current smoker	6 (6.0)	3 (3.2)	1 (1.8)	2 (0.9)
Charlson comorbidity index, *n* (%)				
0	53 (39.8)	32 (45.1)	12 (29.3)	9 (42.9)
1	50 (37.6)	26 (52.0)	18 (36.0)	6 (28.6)
≥ 2	30 (22.6)	13 (18.3)	11 (26.8)	6 (28.6)
APOE4 carrier, *n* (%)	59 (44.3)	17 (23.9)	29 (70.7)	13 (61.9)
Mini-Mental State Examination score, median (IQR)	27.0 (24.0 to 28.0)	28.0 (27.0 to 29.0)	26.0 (24.0 to 28.0)	21.0 (16.0 to 24.0)
Cognitive measure, composite *z* score, mean (SD)				
SNSB attention score	− 0.32 (0.96)	− 0.01 (0.90)	− 0.60 (0.85)	− 0.90 (0.95)
SNSB language and related function score	0.07 (1.49)	0.55 (0.66)	− 0.01 (1.13)	− 1.96 (2.91)
SNSB visuospatial function score	0.21 (2.34)	0.96 (0.72)	0.19 (1.58)	− 2.38 (4.62)
SNSB memory score	− 0.36 (1.70)	0.65 (0.99)	− 0.97 (1.48)	− 2.70 (1.15)
SNSB frontal/executive function score	− 0.26 (2.01)	0.56 (0.78)	− 0.66 (1.02)	− 2.33 (1.43)
Amyloid PET, standard uptake value ratio, mean (SD)	1.16 (0.29)	1.06 (0.30)	1.25 (0.25)	1.35 (0.23)
Hippocampal volume, cm^3^, mean (SD)	7.25 (1.28)	7.67 (0.95)	7.04 (1.43)	6.18 (1.26)

*Abbreviations*: *AD* Alzheimer’s disease, *CN* Cognitively normal, *IQR* Interquartile range, *MCI* Mild cognitive impairment, *SD* Standard deviation, *SNSB* Seoul Neuropsychological Screening Battery, *APOE4* apolipoprotein E, *PET* Positron emission tomography

^a^The diagnostic criteria for MCI were based on the Jak/Bondi comprehensive criteria

Table 2 summarizes the comparison of the classification results of our ensemble machine learning models and the previous statistical approach using the accuracy metrics of the AUC, sensitivity, and specificity. Regarding the classification results of MCI-AD dementia and CN, our proposed machine learning model outperformed the statistical approach for both datasets. In the previous trial, the AUC value from our proposed machine learning model (0.925) was higher than that from the statistical approach (0.873). Similarly, from the additional external trial, the AUC value from our proposed machine learning model (0.825) was higher than that from a statistical approach (0.639). The results indicated that the statistical approach had a limitation of performance bias depending on the statistical value of the data being analyzed (previous trial data only). In contrast, our model minimized the overfitting issue and exhibited the performance of the generalized model. Regarding the classification results of MCI and CN, our proposed machine learning model also outperformed the statistical approach for both datasets. In the previous trial, the AUC value from our proposed machine learning model (0.860) was slightly higher than that from the statistical approach (0.852). From the additional external trial, the AUC value from our proposed machine learning model (0.854) was significantly higher than that from the statistical approach (0.688). The results also indicated that the statistical approach had a limitation of performance bias depending only on the statistical value from the previous trial. However, our model also minimized the overfitting issue by providing similar AUC values for both datasets.

**Table 2:** C-statistic for the prediction model in the diagnosis of AD and MCI

Olfactory-stimulated oxygenation difference in the orbitofrontal cortex	Previous trial (*n* = 97)			Additional trial (*n* = 36)		
	AUC (95% CI)	Sensitivity (%)	Specificity (%)	AUC (95% CI)	Sensitivity (%)	Specificity (%)
Classic prediction model for AD and MCI	0.873 (0.800 to 0.945)	88.1	81.8	0.639 (0.482 to 0.796)	60.0	68.8
Prediction model for AD and MCI using machine learning algorithm	0.925	88.1	80.0	0.825	65.0	81.3
Classic prediction model for MCI (excluded patients with AD)^a^	0.852 (0.764 to 0.939)	84.6	81.8	0.688 (0.527 to 0.848)	68.8	68.8
Prediction model for MCI using machine learning algorithm (excluded patients with AD)^a^	0.860	88.7	81.8	0.854	66.7	81.3

*Abbreviations*: *AD* Alzheimer’s disease, *AUC* Area under the receiver operating characteristic curve, *CI* Confidence interval, *CN* Cognitively normal, *MCI* Mild cognitive impairment

^a^We excluded 16 patients with AD; therefore, the sample size for this analysis was 112

**Fig. 2:**
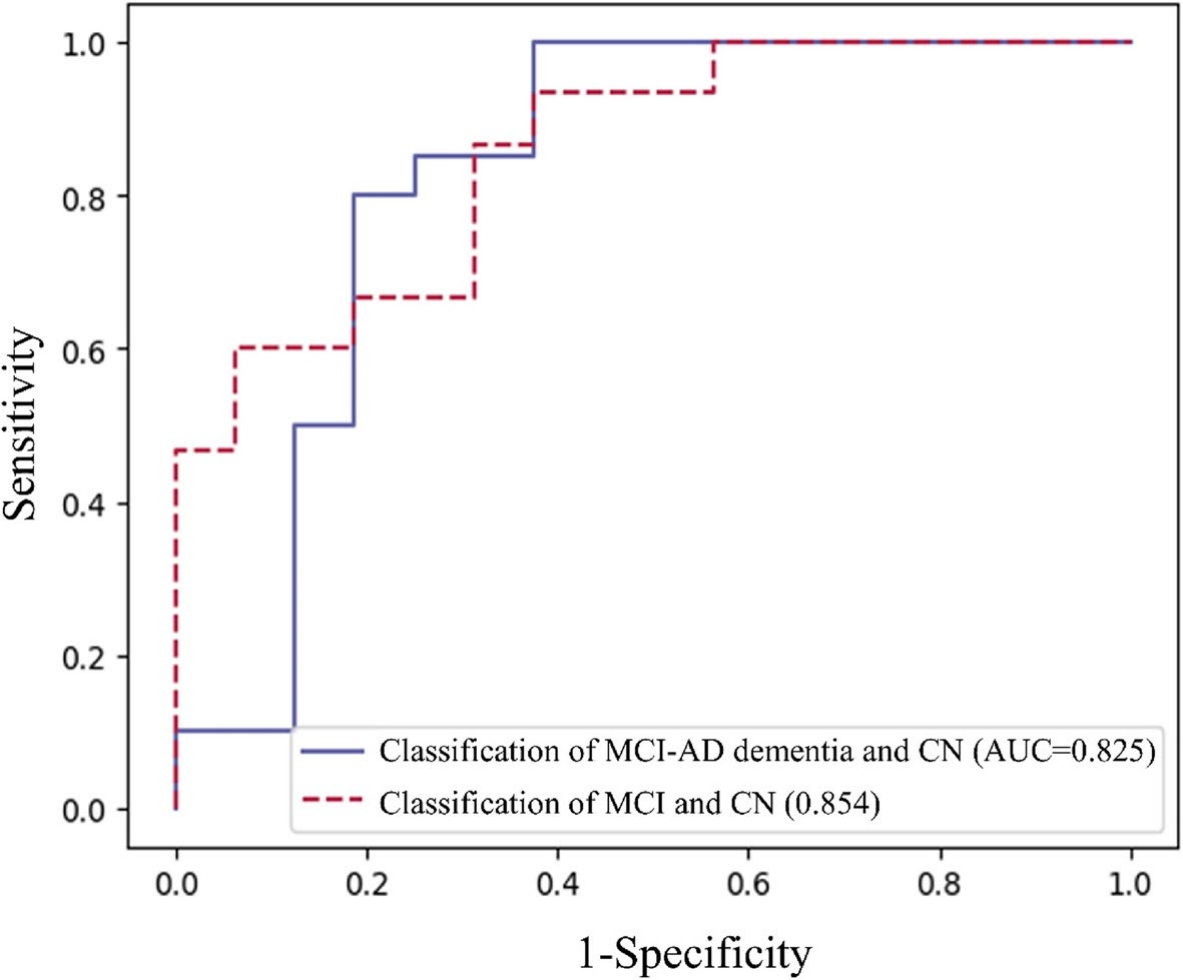
Receiver operating characteristic curve of our models: the classification of MCI-AD and CN and the classification of MCI and CN. CN, cognitively normal; MCI, mild cognitive impairment; AD, Alzheimer’s disease

Figure 2 shows the comparison of receiver operating characteristic curves from the additional external trial data when we considered the machine learning models: our ensemble model and each single machine learning model for the classification of MCI-AD dementia and CN in The results showed that the ensemble model provided higher AUC values in both the classification of MCI-AD dementia and CN and the classification of MCI and CN. We also compared hyper-parameter tuning using grid search and optuna (Fig. S1). More detailed accuracy results for the comparison are shown in Tables S2 and 3 (classification of MCI-AD dementia and CN) and Tables S4 and 5 (classification of MCI and CN).

**Fig. 3:**
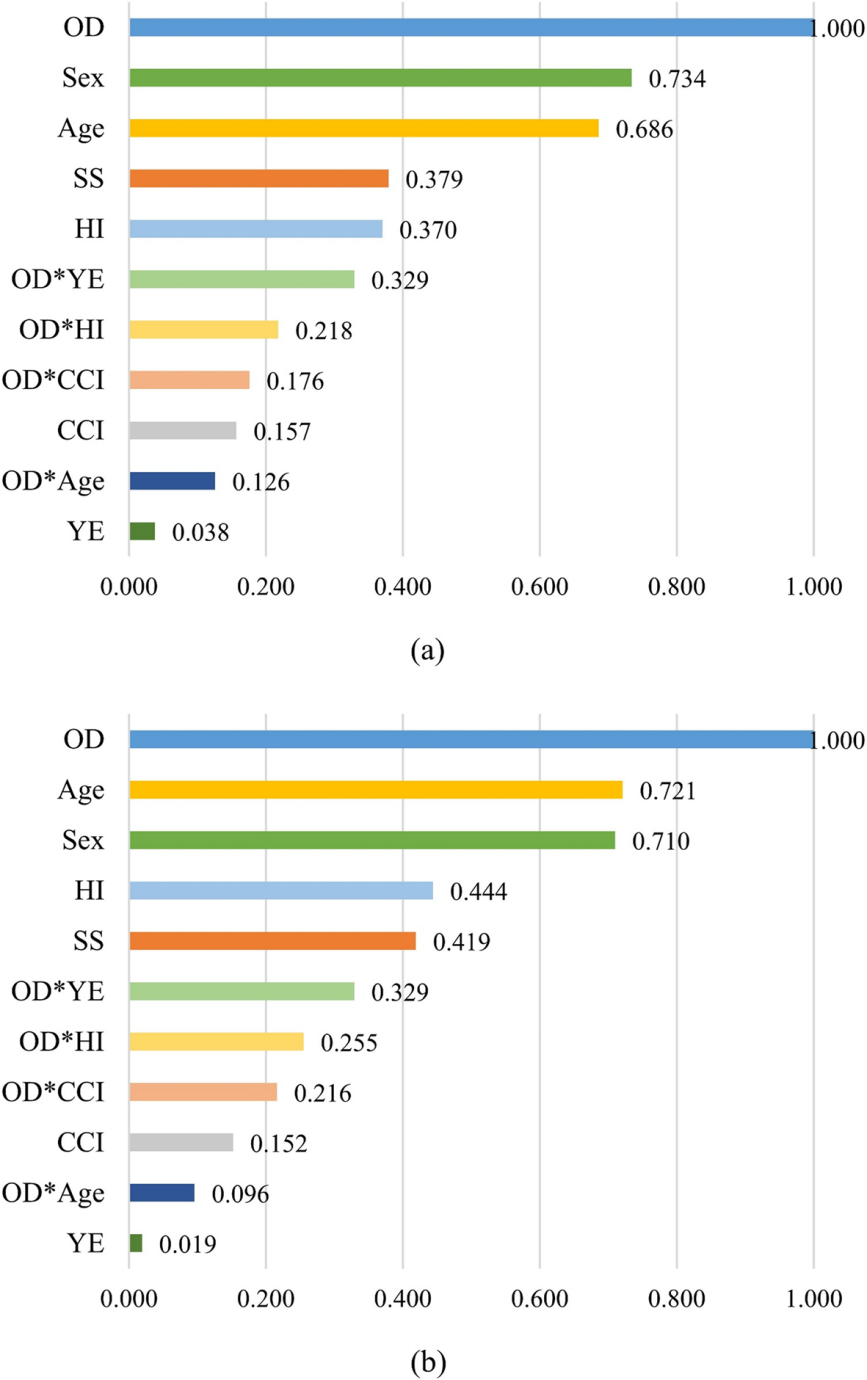
Ranked feature importance values from all 11 features: **a** the classification of MCI-AD and CN and **b** the classification of MCI and CN. Numbers in bold indicate statistically significant associations (*P* < 0.05). CN, cognitively normal; MCI, mild cognitive impairment; AD, Alzheimer’s disease; OD, olfactory-stimulated oxygenation difference in the orbitofrontal cortex; SS, smoking status; HI, household income; YE, years of education; CCI, Charlson comorbidity index

Figure 3a and b show the feature importance values calculated on 11 features for classification of MCI-AD dementia and CN and classification of MCI and CN respectively. For the classification of MCI-AD dementia and CN, fNIRS (1.000) had the highest importance value, followed by sex (0.734), age (0.686), and smoking status (0.379). For the classification of MCI and CN, fNIRS (1.000) had also the highest importance value, followed by age (0.721), sex (0.710), and household income (0.444). The results indicated that fNIRS was the top contributor for both classification models. The feature importance values from the fNIRS were greater than those from age. In addition, we summarized the relationship between the number of features and the performance of the model in Fig. S2 and Table S6. However, the Charlson comorbidity index and fNIRS × age and years of education rarely contributed to both classification models.

## Discussion

### Main findings

Through two independent trials, we found that machine learning models using olfactory-stimulated oxygenation differences in the orbitofrontal cortex were superior in diagnosing MCI and AD dementia compared to the classic statistical model. In this study, we presented two machine learning models for the classification of MCI-AD dementia and CN and for the classification of MCI and CN. Our models used an ensemble approach to combine state-of-the-art models.

For the classification of MCI-AD dementia and CN, we combined four models, the GB and LGB, which provided AUC values of 0.925 and 0.825 for the original and additional trial datasets, respectively. For the classification of MCI and CN, we utilized single model LGB, which provided AUC values of 0.860 and 0.854 for the original and additional trial data, respectively. Compared to the classic statistical approach published in the previous study, our model provided consistent performance regardless of different datasets and higher AUC values. In particular, fNIRS, which is a useful diagnostic method, is the top contributor for both classification AI-driven models. Our results provide quantification of cognitive impairment (MCI and/or AD dementia) using olfactory-stimulated fNIRS with machine learning to improve generalization and reproducibility.

### Comparison with previous studies

Previous studies classifying AD stages using a novel diagnostic method and machine learning investigated wearable EEG (*n* = 26) [22], eye-tracking (*n* = 210) [23], and various genetic or serum biomarkers [24]. However, previous studies have provided little evidence due to the small sample size, lack of an extra-validation dataset, lack of reported feature importance, and use of an observation study dataset [22–24]. In contrast, our study used a novel diagnostic method to identify AD dementia and/or MCI using various AI-driven algorithms and compared them individually through two independent diagnostic trials. Additionally, in a previous study, some of the existing cognitive function tests were studied with the feature values of machine learning [25], which have the potential to distort the results. In fact, a previous study reported that the cognitive function test result had the greatest influence on the model in feature importance [26]. To solve this problem, the model was trained using only fNIRS, sex, years of education, age, smoking status, and the Charlson comorbidity index. In addition, the covariate problem was solved by adjusting each of the continuous variables, such as age, years of education, household income, and Charlson comorbidity index, which could affect the fNIRS data. In terms of the feature importance of our model, the fNIRS value was found to be the most effective for identifying AD dementia and/or MCI.

### Possible explanations for our results

This study was conducted in real-world practice using original and independent additional trials. Classic statistical methods cannot guarantee generalization and reproducibility in real-world practice. However, AI-driven machine learning can solve these limitations by using variable pruning and group improvement.

The fNIRS system can continuously measure changes in the concentrations of oxidized hemoglobin and reduced hemoglobin in the cerebral cortex, making it a suitable system for tracking cerebral activity indicators [27, 28]. In this respect, it has several advantages over imaging equipment, such as functional MRI and amyloid PET. First, there is no problem of radiation exposure as with amyloid PET or brain CT, and there is no need to place the patient in a narrow place as with MRI. Second, olfactory-stimulated fNIRS is faster (3 min) to perform than the SNSB (90 min or more), which is essential for the diagnosis of AD [29]. Our novel method is easily accessible to people who are illiterate or not cooperative with AD-related examinations. Third, this method is much cheaper than brain MRI and amyloid PET, making it easily accessible, even in underdeveloped countries. Finally, this novel method enables the rapid diagnosis of MCI; thus, it is possible to provide more precise medical services to patients with MCI to prevent AD dementia.

### Policy implication

Validation of our diagnostic method through machine learning can provide stable accuracy even when applied to new patient populations, especially illiterate patients who are difficult to diagnose using questionnaires, in addition to low cost, low patient risk (i.e., radiation risk), and short diagnostic time (3 min). We believe that these algorithms can also be installed on mobile devices, allowing them to perform cognitive function assessments beyond the limitations of patients who cannot see experts in person [7]. This can help address the medical disparities between low-income and high-income patients, urban and rural areas, and developed and developing countries.

### Strengths and limitations

This study has some limitations. First, although we recruited and included additional patients in an independent trial, it was still a small Asian population. Therefore, it is necessary to verify the results through an international, large-scale trial. Comprehensive longitudinal studies are required. Second, our study performed brain MRI, amyloid PET, and *APOE4* genotyping in patients, but these data were not analyzed because they were not suitable for the purpose of our study. Further research is needed to determine the potential relationship between olfactory-stimulated fNIRS and the aforementioned data. Finally, there is a need for early intervention efforts in patients diagnosed with MCI using our novel methodology. Thus, policy and cost-effectiveness studies on the early prevention of AD among patients with MCI are warranted [30].

Despite these limitations, this study’s findings are meaningful. We found that the machine learning model achieved a high level of external validation accuracy in several algorithms. Moreover, our proposed machine learning method showed high accuracy and stability compared with statistical linear models in external validation. Therefore, our results suggest that this novel method can be a potential indicator for identifying cognitive impairments, such as AD dementia and/or MCI.

## Conclusions

This is the first study to apply machine learning and statistical models to recruit patients for external validation of the olfactory-stimulated fNIRS diagnostic technique using a previous statistical model. Through two independent trials, we found that machine learning models using olfactory-stimulated oxygenation differences in the orbitofrontal cortex were superior in diagnosing MCI and/or AD dementia compared to the classic statistical model. Our results suggest that the machine learning algorithm is stable across different patient groups and increases generalization and reproducibility. We suggest that this machine learning model with a novel fNIRS approach can be used as a potential diagnostic tool for patients with MCI and/or AD dementia.

## Supplementary Information

**Additional file 1: Figure S1.** The changes in AUROC with an increase in the number of features: (a) the classification of MCI-AD and CN and (b) the classification of MCI and CN. **Figure S2.** SHAP values from all 11 features: (a) the classification of MCI-AD and CN and (b) the classification of MCI and CN. **Table S1.** Baseline characteristics of participants at enrollment (additional trial *n* = 36). **Table S2.** The changes in accuracy with an increase in the number of features were examined in the MCI-AD dementia and CN groups. **Table S3.** The changes in accuracy with an increase in the number of features were examined in the MCI and CN groups. **Table S4.** Results of stacking techniques applied to ensemble-based models for MCI-AD and CN classification. **Table S5.** Mini-Mental State Examination for correlation with the AD diagnosis.

## Data Availability

The authors confirm that data supporting the findings of this study are available upon reasonable request.

## References

[1] Rasmussen J, Langerman H (2019). Alzheimer’s disease - why we need early diagnosis. Degener Neurol Neuromuscul Dis.

[2] Fisher CK, Smith AM, Walsh JR (2019). Machine learning for comprehensive forecasting of Alzheimer’s Disease progression. Sci Rep.

[3] Zhang J, Zhao Z, Sun S, Li J, Wang Y, Dong J, Yang S, Lou Y, Yang J, Li W (2022). Olfactory evaluation in Alzheimer’s disease model mice. Brain Sci.

[4] Son G, Jahanshahi A, Yoo SJ, Boonstra JT, Hopkins DA, Steinbusch HWM, Moon C (2021). Olfactory neuropathology in Alzheimer’s disease: a sign of ongoing neurodegeneration. BMB Rep.

[5] O’Connor A, Cash DM, Poole T, Markiewicz PJ, Fraser MR, Malone IB, Jiao J, Weston PSJ, Flores S, Hornbeck R (2023). Tau accumulation in autosomal dominant Alzheimer’s disease: a longitudinal [(18)F]flortaucipir study. Alzheimers Res Ther.

[6] Turri M, Conti E, Pavanello C, Gastoldi F, Palumbo M, Bernini F, Aprea V, Re F, Barbiroli A, Emide D (2023). Plasma and cerebrospinal fluid cholesterol esterification is hampered in Alzheimer’s disease. Alzheimers Res Ther.

[7] Kim J, Yon DK, Choi KY, Lee JJ, Kim N, Lee KH, Kim JG (2022). Novel diagnostic tools for identifying cognitive impairment using olfactory-stimulated functional near-infrared spectroscopy: patient-level, single-group, diagnostic trial. Alzheimers Res Ther.

[8] Kim J, Kim SC, Kang D, Yon DK, Kim JG (2022). Classification of Alzheimer's disease stage using machine learning for left and right oxygenation difference signals in the prefrontal cortex: a patient-level, single-group, diagnostic interventional trial. Eur Rev Med Pharmacol Sci.

[9] Yoo IK, Marshall DC, Cho JY, Yoo HW, Lee SW (2021). N-Nitrosodimethylamine-contaminated ranitidine and risk of cancer in South Korea: a nationwide cohort study. Life Cycle.

[10] Chin J, Park J, Yang SJ, Yeom J, Ahn Y, Baek MJ, Ryu HJ, Lee BH, Han NE, Ryu KH (2018). Re-standardization of the Korean-Instrumental Activities of Daily Living (K-IADL): clinical usefulness for various neurodegenerative diseases. Dement Neurocogn Disord.

[11] Ahn HJ, Chin J, Park A, Lee BH, Suh MK, Seo SW, Na DL (2010). Seoul Neuropsychological Screening Battery-dementia version (SNSB-D): a useful tool for assessing and monitoring cognitive impairments in dementia patients. J Korean Med Sci.

[12] McKhann GM, Knopman DS, Chertkow H, Hyman BT, Jack CR, Kawas CH, Klunk WE, Koroshetz WJ, Manly JJ, Mayeux R (2011). The diagnosis of dementia due to Alzheimer’s disease: recommendations from the National Institute on Aging-Alzheimer’s Association workgroups on diagnostic guidelines for Alzheimer’s disease. Alzheimer’s Dementia.

[13] Jak AJ, Bondi MW, Delano-Wood L, Wierenga C, Corey-Bloom J, Salmon DP, Delis DC (2009). Quantification of five neuropsychological approaches to defining mild cognitive impairment. Am J Geriatr Psychiatry.

[14] Metzger FG, Schopp B, Haeussinger FB, Dehnen K, Synofzik M, Fallgatter AJ, Ehlis AC (2016). Brain activation in frontotemporal and Alzheimer's dementia: a functional near-infrared spectroscopy study. Alzheimer’s Res Ther.

[15] Yon DK, Lee SW, Ha EK, Lee KS, Jung YH, Jee HM, Kim MA, Ahn JC, Sheen YH, Han MY (2018). Serum lipid levels are associated with allergic rhinitis, nasal symptoms, peripheral olfactory function, and nasal airway patency in children. Allergy.

[16] Lee SW (2022). Methods for testing statistical differences between groups in medical research: statistical standard and guideline of Life Cycle Committee. Life Cycle.

[17] Lee SW (2022). Regression analysis for continuous independent variables in medical research: statistical standard and guideline of Life Cycle Committee. Life Cycle.

[18] Chung H, Ko H, Lee H, Yon DK, Lee WH, Kim TS, Kim KW, Lee J (2023). Development and validation of a deep learning model to diagnose COVID-19 using time-series heart rate values before the onset of symptoms. J Med Virol.

[19] Lee SW, Yang JM, Moon SY, Kim N, Ahn YM, Kim JM, Shin JI, Suh DI, Yon DK (2021). Association between mental illness and COVID-19 in South Korea: a post-hoc analysis. Lancet Psychiatry.

[20] Lee SW, Yang JM, Yoo IK, Moon SY, Ha EK, Yeniova A, Cho JY, Kim MS, Shin JI, Yon DK (2021). Proton pump inhibitors and the risk of severe COVID-19: a post-hoc analysis from the Korean nationwide cohort. Gut.

[21] Cha H, Kim S, Son Y (2021). Associations between cognitive function, depression, and olfactory function in elderly people with dementia in Korea. Front Aging Neurosci.

[22] Perez-Valero E, Lopez-Gordo M, Gutiérrez CM, Carrera-Muñoz I, Vílchez-Carrillo RM (2022). A self-driven approach for multi-class discrimination in Alzheimer’s disease based on wearable EEG. Comput Methods Programs Biomed.

[23] Sun J, Liu Y, Wu H, Jing P, Ji Y (2022). A novel deep learning approach for diagnosing Alzheimer’s disease based on eye-tracking data. Front Hum Neurosci.

[24] Lin RH, Wang CC, Tung CW (2022). A machine learning classifier for predicting stable MCI patients using gene biomarkers. Int J Environ Res Public Health.

[25] Tohka J, Moradi E, Huttunen H (2016). Comparison of feature selection techniques in machine learning for anatomical brain MRI in dementia. Neuroinformatics.

[26] Khajehpiri B, Moghaddam HA, Forouzanfar M, Lashgari R, Ramos-Cejudo J, Osorio RS, Ardekani BA (2022). Survival analysis in cognitively normal subjects and in patients with mild cognitive impairment using a proportional hazards model with extreme gradient boosting regression. J Alzheimer’s Dis.

[27] Ehlis AC, Schneider S, Dresler T, Fallgatter AJ (2014). Application of functional near-infrared spectroscopy in psychiatry. Neuroimage.

[28] Gossé LK, Bell SW, Hosseini SMH (2022). Functional near-infrared spectroscopy in developmental psychiatry: a review of attention deficit hyperactivity disorder. Eur Arch Psychiatry Clin Neurosci.

[29] Salvatore C, Cerasa A, Castiglioni I (2018). MRI characterizes the progressive course of AD and predicts conversion to Alzheimer's dementia 24 months before probable diagnosis. Front Aging Neurosci.

[30] Lee YS, Youn H, Jeong HG, Lee TJ, Han JW, Park JH, Kim KW (2021). Cost-effectiveness of using amyloid positron emission tomography in individuals with mild cognitive impairment. Cost Eff Res Alloc.

