# Supplementary material for: Quantification of identifying cognitive impairment using olfactory-stimulated functional near-infrared spectroscopy with machine learning: a post hoc analysis of a diagnostic trial and validation of an external additional trial
Source: Alzheimers Res Ther. 2023 Jul 22;15:127. doi: 10.1186/s13195-023-01268-9 (PMC10362671; doi:10.1186/s13195-023-01268-9)
Supplement: Supplementary file 1 — Additional file 1: Figure S1. The changes in AUROC with an increase in the number of features: (a) the classification of MCI-AD and CN and (b) the classification of MCI and CN. Figure S2. SHAP values from all 11 features: (a) the classification of MCI-AD and CN and (b) the classification of MCI and CN. Table S1. Baseline characteristics of participants at enrollment (additional trial n = 36). Table S2. The changes in accuracy with an increase in the number of features were examined in the MCI-AD dementia and CN groups. Table S3. The changes in accuracy with an increase in the number of features were examined in the MCI and CN groups. Table S4. Results of stacking techniques applied to ensemble-based models for MCI-AD and CN classification. Table S5. Mini-Mental State Examination for correlation with the AD diagnosis. [file 13195_2023_1268_MOESM1_ESM.docx]

**Quantification of identifying cognitive impairment using olfactory-stimulated functional near-infrared spectroscopy with machine learning: a post-hoc analysis of a diagnostic trial and validation of an external additional trial**

Jaewon Kim, MD, PhD^a†^, Hayeon Lee, MS^b†^, Jinseok Lee, PhD^b^, Sang Youl Rhee, MD, PhD^a^, Jae Il Shin, MD, PhD^d^, Seung Won Lee, MD, PhD^e^, Wonyoung Cho, PhD^a^, Chanyang Min, PhD^a^, Rosie Kwon, MS^a,f^, Jae Gwan Kim, PhD^g^**^*^**, Dong Keon Yon, MD, FACAAI, FAAAAI^a,f^**^*^**

| **List of Supporting Information** |
| --- |

**Figure S1**. The changes in AUROC with an increase in the number of features: (**a**) the classification of MCI-AD and CN and (**b**) the classification of MCI and CN

**Figure S2.** SHAP values from all 11 features: (**a**) the classification of MCI-AD and CN and (**b**) the classification of MCI and CN

**Table S1.** Baseline characteristics of participants at enrollment (additional trial n=36).

**Table S2.** The changes in accuracy with an increase in the number of features were examined in the MCI-AD dementia and CN groups.

**Table S3.** The changes in accuracy with an increase in the number of features were examined in the MCI and CN groups.

**Table S4.** Results of stacking techniques applied to ensemble-based models for MCI-AD and CN classification.

**Table S5.** Mini-Mental State Examination for correlation with the AD diagnosis

**Figure S1**. The changes in AUROC with an increase in the number of features: (**a**) the classification of MCI-AD and CN and (**b**) the classification of MCI and CN


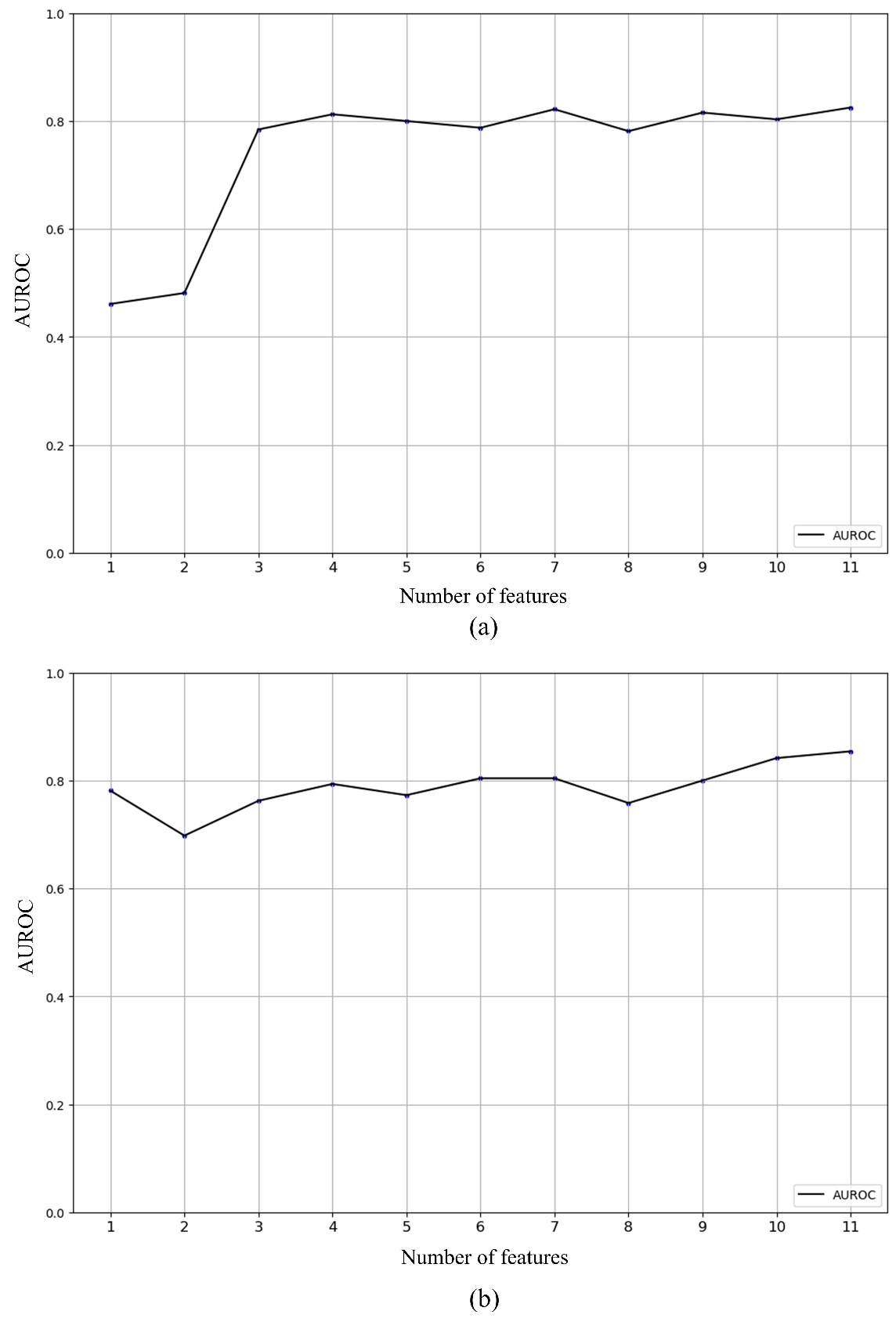


**Figure S2.** SHAP values from all 11 features: (**a**) the classification of MCI-AD and CN and (**b**) the classification of MCI and CN.

Numbers in bold indicate statistically significant associations (P<0.05).

CN, cognitively normal; MCI, mild cognitive impairment; AD, Alzheimer disease; OD, olfactory-stimulated oxygenation difference in the orbitofrontal cortex; SS, smoking status; HI, household income; YE, years of education; CCI, Charlson comorbidity index.

**
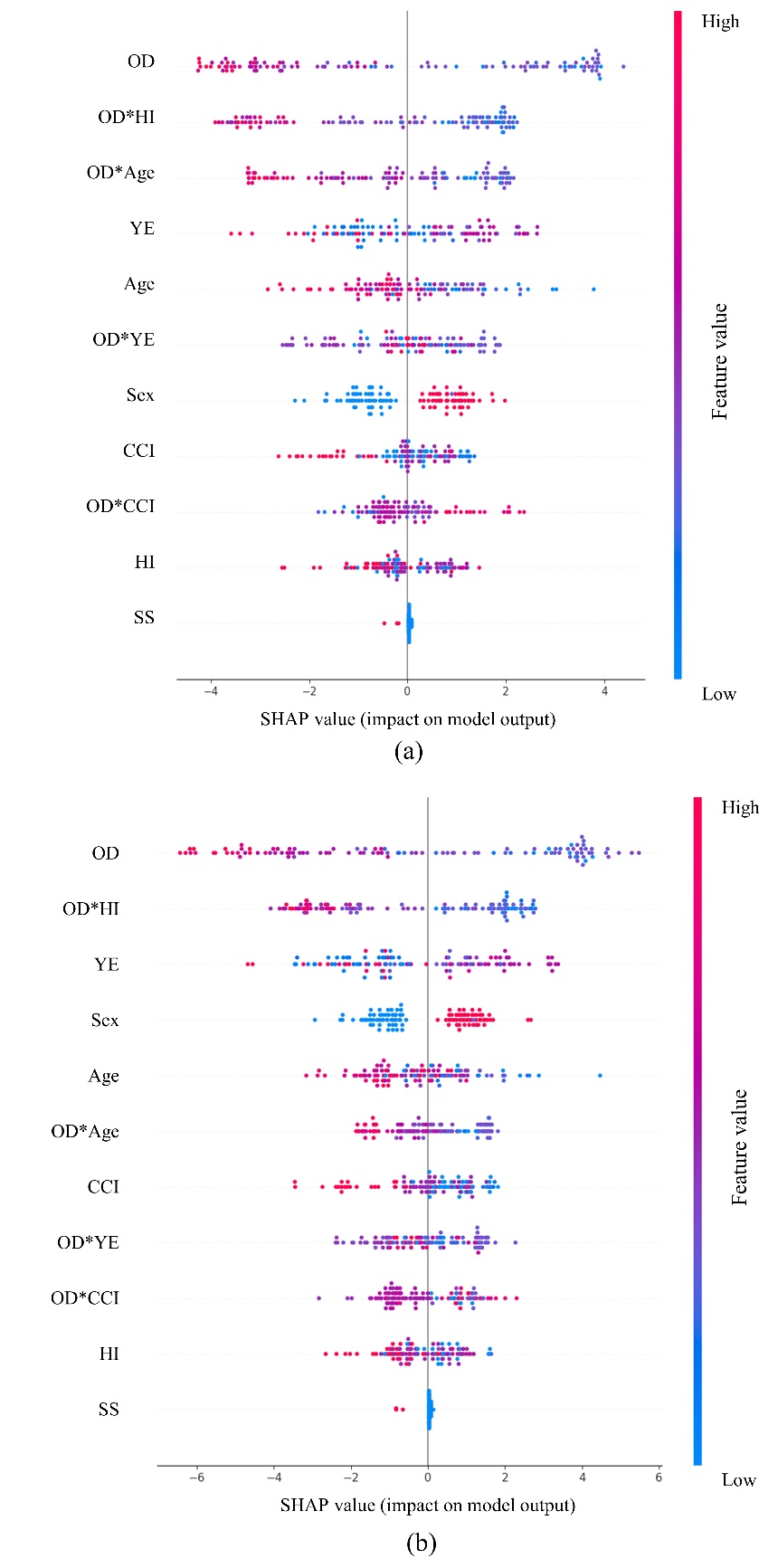
**

**Table S1.** Baseline characteristics of participants at enrollment (additional trial n=36).

|  | CN | MCI^a^ | AD dementia |
| --- | --- | --- | --- |
| Number (%) | 16 | 15 | 5 |
| Age, years, median (IQR) | 75.0 (72.3 to 77.8) | 72.0 (69.0 to 76.0) | 76.0 (68.0 to 83.0) |
| Body mass index, kg/m^2^, n (%) |  |  |  |
| <25 (normal) | 11 (68.8) | 7 (46.7) | 4 (80.0) |
| ≥25 (overweight or obese) | 5 (31.3) | 8 (53.3) | 1 (20.0) |
| Sex, female (%) | 8 (50.0) | 9 (60.0) | 3 (60.0) |
| Education, years, median (IQR) | 11.0 (9.0 to 16.0) | 12.0 (6.0 to 14.0) | 12.0 (3.5 to 14.0) |
| Household income, n (%) |  |  |  |
| Low (1–29 percentile) | 3 (18.8) | 4 (26.7) | 1 (20.0) |
| Middle (30–69 percentile) | 6 (37.5) | 8 (53.3) | 3 (60.0) |
| High (70–100 percentile) | 7 (43.8) | 3 (20.0) | 1 (20.0) |
| Smoking status, n (%) |  |  |  |
| Never or ex-smoker | 16 (100.0) | 14 (93.3) | 5 (100.0) |
| Current smoker | 0 (0.0) | 1 (6.7) | 0 (0.0) |
| Charlson comorbidity index, n (%) |  |  |  |
| 0 | 12 (75.0) | 3 (30.0) | 3 (60.0) |
| 1 | 2 (12.5) | 7 (46.7) | 1 (20.0) |
| ≥2 | 2 (12.5) | 5 (33.3) | 1 (20.0) |
| APOE4 carrier, n (%) | 3 (18.8) | 11 (73.3) | 2 (40.0) |
| Mini-Mental State Examination score, median (IQR) | 28.0 (28.0 to 29.8) | 26.0 (24.0 to 29.0) | 21.0 (16.5 to 24.0) |
| Cognitive measure, composite z score, mean (SD) |  |  |  |
| SNSB attention score | 0.22 (0.84) | -0.74 (0.95) | -1.73 (0.27) |
| SNSB language and related function score | 0.70 (0.68) | -0.06 (0.99) | -1.13 (0.85) |
| SNSB visuospatial function score | 0.79 (0.87) | 0.23 (1.42) | -2.17 (3.74) |
| SNSB memory score | 0.38 (0.56) | -1.54 (1.38) | -2.59 (0.62) |
| SNSB frontal/executive function score | 0.57 (0.73) | -0.97 (0.87) | -2.20 (1.23) |
| Amyloid PET, standard uptake value ratio, mean (SD) | 0.49 (0.54) | 1.18 (0.22) | 1.00 (0.13) |
| Hippocampal volume, cm^3^, mean (SD) | 7.63 (0.86) | 7.11 (1.53) | 6.58 (1.28) |

Abbreviations: AD, Alzheimer disease; CN, cognitively normal; IQR, interquartile range; MCI, mild cognitive impairment; SD, standard deviation; SNSB, Seoul Neuropsychological Screening Battery; APOE4, apolipoprotein E; PET, positron emission tomography.

^a^ The diagnostic criteria for MCI were based on the Jak/Bondi comprehensive criteria.

**Table S2.** The changes in accuracy with an increase in the number of features were examined in the MCI-AD dementia and CN groups.

|  | Previous trial (n=97) | | | Additional trial (n=36) | | |
| --- | --- | --- | --- | --- | --- | --- |
| **Feature, n** | **AUC** | **sensitivity** | **specificity** | **AUC** | **sensitivity** | **specificity** |
| 1 | 0.4072$\pm$0.1631 | 0.8111$\pm$0.1238 | 0.2000$\pm$0.2400 | 0.4609 | 0.3500 | 0.4375 |
| 2 | 0.3770$\pm$0.1212 | 0.8833$\pm$0.0706 | 0.0909$\pm$0.1150 | 0.4813 | 0.4000 | 0.4375 |
| 3 | 0.8682$\pm$0.0847 | 0.8861$\pm$0.1218 | 0.7818$\pm$0.1233 | 0.7844 | 0.5500 | 0.8125 |
| 4 | 0.8629$\pm$0.0724 | 0.8611$\pm$0.1079 | 0.8000$\pm$0.1336 | 0.8125 | 0.5500 | 0.8125 |
| 5 | 0.8727$\pm$0.0981 | 0.8583$\pm$0.0889 | 0.7636$\pm$0.1233 | 0.8000 | 0.6000 | 0.8125 |
| 6 | 0.8864$\pm$0.0843 | 0.8583$\pm$0.0889 | 0.7636$\pm$0.1233 | 0.7875 | 0.6500 | 0.8125 |
| 7 | 0.8859$\pm$0.0646 | 0.8583$\pm$0.0889 | 0.7455$\pm$0.1060 | 0.8219 | 0.6000 | 0.8125 |
| 8 | 0.8970$\pm$0.0699 | 0.8583$\pm$0.0889 | 0.7636$\pm$0.1233 | 0.7813 | 0.6000 | 0.8125 |
| 9 | 0.8922$\pm$0.0628 | 0.8806$\pm$0.1060 | 0.7455$\pm$0.1060 | 0.8156 | 0.6000 | 0.8125 |
| 10 | 0.8773$\pm$0.0577 | 0.8583$\pm$0.0889 | 0.7455$\pm$0.1060 | 0.8031 | 0.5500 | 0.8125 |
| 11 | 0.9253$\pm$0.0461 | 0.8806$\pm$0.1060 | 0.8000$\pm$0.1336 | 0.8250 | 0.6500 | 0.8125 |

Abbreviations: AUC, area under the receiver operating characteristic curve; CN, cognitively normal; MCI, mild cognitive impairment; AD, Alzheimer disease.

**Table S3.** The changes in accuracy with an increase in the number of features were examined in the MCI and CN groups.

|  | Previous trial (n=81) | | | Additional trial (n=31) | | |
| --- | --- | --- | --- | --- | --- | --- |
| **Feature, n** | **AUC** | **sensitivity** | **specificity** | **AUC** | **sensitivity** | **specificity** |
| 1 | 0.8073$\pm$0.1323 | 0.9200$\pm$0.0980 | 0.7091$\pm$0.1854 | 0.7813 | 0.6000 | 0.8125 |
| 2 | 0.6455$\pm$0.1054 | 0.6133$\pm$0.1809 | 0.6909$\pm$0.1477 | 0.6979 | 0.4667 | 0.8125 |
| 3 | 0.8273$\pm$0.1004 | 0.8113$\pm$0.1655 | 0.7455$\pm$0.1666 | 0.7625 | 0.7333 | 0.8125 |
| 4 | 0.8691$\pm$0.0879 | 0.9933$\pm$0.2255 | 0.7455$\pm$0.1564 | 0.7938 | 0.7333 | 0.7500 |
| 5 | 0.8664$\pm$0.1055 | 0.8467$\pm$0.1485 | 0.7818$\pm$0.1686 | 0.7729 | 0.6000 | 0.7500 |
| 6 | 0.8642$\pm$0.1340 | 0.8067$\pm$0.2195 | 0.7636$\pm$0.1686 | 0.8012 | 0.6667 | 0.8125 |
| 7 | 0.8642$\pm$0.1340 | 0.8067$\pm$0.2195 | 0.7636$\pm$0.1686 | 0.8042 | 0.6667 | 0.8125 |
| 8 | 0.8539$\pm$0.0990 | 0.8067$\pm$0.1272 | 0.7818$\pm$0.1686 | 0.7583 | 0.6667 | 0.8125 |
| 9 | 0.8333$\pm$0.1302 | 0.7667$\pm$0.0843 | 0.7818$\pm$0.1686 | 0.8000 | 0.6000 | 0.8125 |
| 10 | 0.8606$\pm$0.0809 | 0.8267$\pm$0.2736 | 0.8000$\pm$0.1336 | 0.8417 | 0.6667 | 0.8125 |
| 11 | 0.8600$\pm$0.0800 | 0.8867$\pm$0.1572 | 0.8182$\pm$0.1408 | 0.8542 | 0.6667 | 0.8125 |

Abbreviations: AUC, area under the receiver operating characteristic curve; CN, cognitively normal; MCI, mild cognitive impairment.

**Table S4.** Results of stacking techniques applied to ensemble-based models for MCI-AD and CN classification.

|  | Previous trial (n=81) | | | Additional trial (n=31) | | |
| --- | --- | --- | --- | --- | --- | --- |
| **model** | **AUC** | **sensitivity** | **specificity** | **AUC** | **sensitivity** | **specificity** |
| SVM | 0.8909$\pm$0.0721 | 0.8806$\pm$0.1060 | 0.8000$\pm$0.1336 | 0.6375 | 0.5500 | 0.8125 |
| XGBoost | 0.8909$\pm$0.0721 | 0.8806$\pm$0.1060 | 0.8000$\pm$0.1336 | 0.6813 | 0.5500 | 0.8125 |
| LGB | 0.8909$\pm$0.0721 | 0.8806$\pm$0.1060 | 0.8000$\pm$0.1336 | 0.7266 | 0.5500 | 0.8125 |
| GB | 0.8909$\pm$0.0721 | 0.8806$\pm$0.1060 | 0.8000$\pm$0.1336 | 0.6813 | 0.5500 | 0.8125 |
| LR | 0.8909$\pm$0.0721 | 0.8806$\pm$0.1060 | 0.8000$\pm$0.1336 | 0.7969 | 0.5500 | 0.8125 |
| Adaboost | 0.8909$\pm$0.0721 | 0.8806$\pm$0.1060 | 0.8000$\pm$0.1336 | 0.6813 | 0.5500 | 0.8125 |

Abbreviations: XGBoost, extreme gradient boosting; GB, gradient boosting; AdaBoost, adaptive boosting; LGB, light gradient boosting; LR, logistic regression; SVM, support vector machine; AUC, area under the receiver operating characteristic curve; CN, cognitively normal; MCI, mild cognitive impairment.

**Table S5.** Mini-Mental State Examination for correlation with the AD diagnosis (regression model)

| Variables | Crude model | | Adjusted model* | |
| --- | --- | --- | --- | --- |
|  | β (95% CI) | P value | β (95% CI) | P value |
| **Mini-Mental State Examination** | **0.144 (0.076 to 0.211)** | **<0.001** | **0.145 (0.077 to 0.212)** | **<0.001** |

* Risk factors were adjusted for dataset (original and posy-hoc cohort,) age, sex, education years (continuous), household income (low, middle, and high), smoking (never or ex-smoker and current smoker), and Charlson comorbidity index (0, 1, and ≥2).

Numbers in bold indicate statistically significant associations (P < 0.05)
